# Impact of Short-Term Training Camp on Aortic Blood Pressure in Collegiate Endurance Runners

**DOI:** 10.3389/fphys.2018.00290

**Published:** 2018-03-28

**Authors:** Tsubasa Tomoto, Jun Sugawara, Ai Hirasawa, Tomoko Imai, Seiji Maeda, Shigehiko Ogoh

**Affiliations:** ^1^Human Informatics Research Institute, National Institute of Advanced Industrial Science and Technology, Tsukuba, Japan; ^2^Faculty of Health Sciences, Kyorin University, Mitaka, Japan; ^3^Center for General Education, Aichi Institute of Technology, Toyota, Japan; ^4^Faculty of Health and Sports Sciences, University of Tsukuba, Tsukuba, Japan; ^5^Department of Biomedical Engineering, Toyo University, Kawagoe, Japan

**Keywords:** aortic blood pressure, pulse wave analysis, endurance training, vigorous training, athletic conditioning

## Abstract

To investigate the influence of short-term vigorous endurance training on aortic blood pressure (BP), pulse wave analysis was performed in 36 highly trained elite collegiate endurance runners before and after a 7-day intense training camp. Subjects participated three training sessions per day, which mainly consisted of long distance running and sprint training to reach the daily target distance of 26 km. After the camp, they were divided into two groups based on whether the target training was achieved. Aortic systolic BP, pulse pressure, and tension-time index (TTI, a surrogate index of the myocardial oxygen demand) were significantly elevated after the camp in the accomplished group but not in the unaccomplished group, whereas the brachial BP remained unchanged in both groups. The average daily training distance was significantly correlated with the changes in aortic systolic BP (*r* = 0.608, *p* = 0.0002), pulse pressure (*r* = 0.415, *p* = 0.016), and TTI (*r* = 0.438, *p* = 0.011). These results suggest that aortic BP is affected by a short-term vigorous training camp even in highly trained elite endurance athletes presumably due to a greater training volume compared to usual.

## Introduction

There is a widely held notion that the central arterial pressure waveform is synthesized by the overlapped reflection waves returning from the periphery (mainly lower body) on the incident wave in phase; therefore, there are disparities between aortic and peripheral BP waveforms (Nichols and McDonald, 2011). Importantly, aortic BP is more strongly related to concentric left ventricular geometry than brachial BP (Roman et al., 2007). Furthermore, systolic BP is strongly related to left ventricular hypertrophy, and pulse pressure is strongly related to vascular stiffening (Roman et al., 2007, 2010). Thus, assessment of aortic BP could be an indicator of cardiac and vascular condition.

The beneficial effects of endurance exercise at a moderate intensity on the cardiovascular system are well recognized (Thompson et al., 2003; Seals et al., 2008; Rowe et al., 2014). On the other hand, the unfavorable effects of cardiovascular system including higher aortic stiffness and aortic BP have been confirmed among highly endurance-trained populations who participate in prolonged intense exercise events, especially marathons running (Scharhag et al., 2005; Vlachopoulos et al., 2010) and ultramarathons running (Knez et al., 2006; Burr et al., 2014) compared with age-matched physically active peers. However, the potential genetic influences in the aforementioned results cannot be completely ruled out due to a cross-sectional study design (Scharhag et al., 2005; Knez et al., 2006; Vlachopoulos et al., 2010; Burr et al., 2014). Vlachopoulos et al. (2010) and Scharhag et al. (2005) observed that peripheral and aortic BP and pulse pressure were significantly reduced 24 h after a marathon running race (Scharhag et al., 2005; Vlachopoulos et al., 2010), but this response seems to reflect post-exercise hypotension (Halliwill, 2001). Thus, the effect of high intense exercise training (e.g., repetition of intense endurance exercise bouts) on the aortic BP and pulse pressure is still unknown.

We previously reported that in well-trained male collegiate endurance runners, systemic arterial stiffness increased after a 7-day intense endurance training camp (Tomoto et al., 2015). Since arterial stiffening promotes early return of the reflected wave from peripheral to the heart and increases aortic BP (Nichols and McDonald, 2011), we hypothesized that the arterial stiffening induced by intense endurance training may cause to amplify the aortic arterial pressure waveform. As a follow-up study, the purpose of this study was to determine the effect of intense endurance exercise bouts to aortic BP.

## Methods

We recruited the subjects from national ranked *Ekiden relay race* team in Japan. We studied a total of 36 well-trained male collegiate endurance runners. The average of their official best times for a 5,000-m race was 14′28″ ± 0′17″ (mean ± SD). All of the subjects were healthy, normotensive (<140/90 mmHg), non-obese (Body mass index, BMI <25 kg/m^2^), nonsmokers, who were free of medication as well as overt chronic heart and lung disease as assessed by their medical histories. None of the subjects were taking cardiovascular-acting medication. This study was reviewed and approved by the Institutional Review Board (Toyo University: 2012-R-04). Additionally, all procedures conformed to the ethical guidelines of the Helsinki Declaration. All subjects provided informed written consent prior to participation.

**Figure 1:**
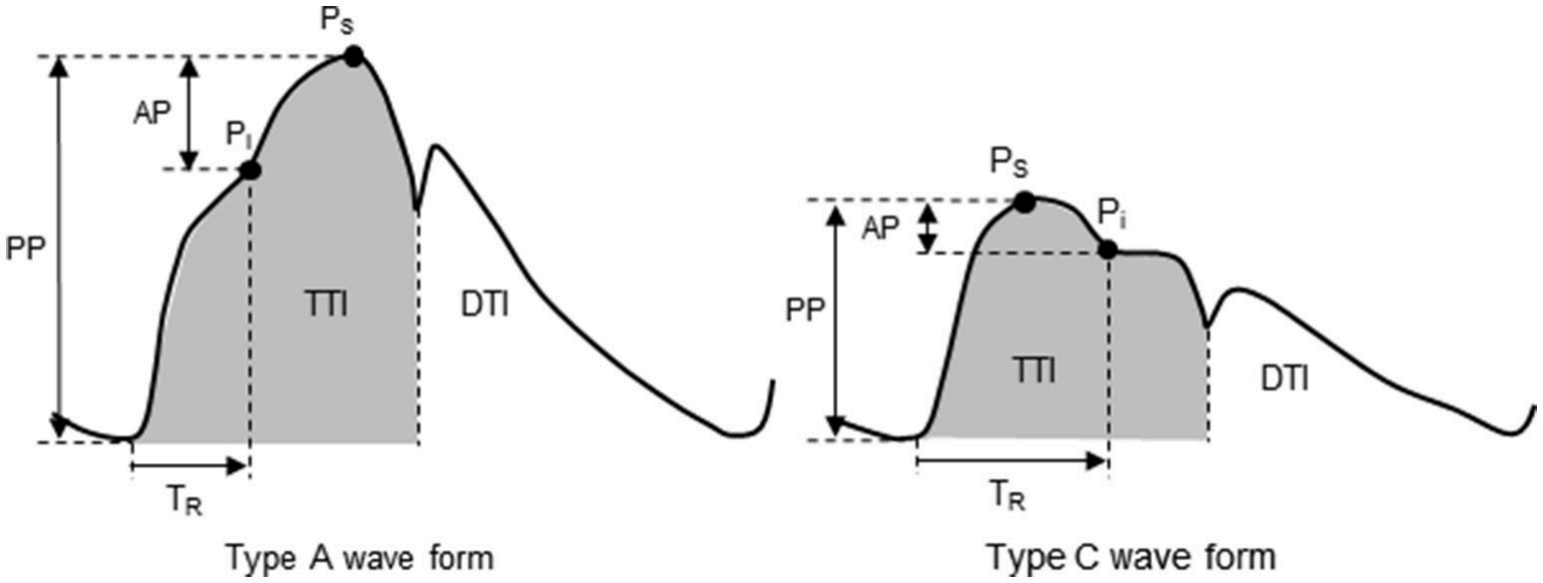
Aortic pulse wave analysis. Type A waveform: reflecting wave during early systole produces an augmented systolic pressure. Type C waveform: reflecting wave during late systole produces a longer T_R_ and Pi after Ps. AP, augmentation pressure; DTI, diastolic tension index; Pi, incident pressure from a reflecting pressure wave; PP, pulse pressure; Ps, systolic pressure; T_R_, round-trip travel time of the reflecting pressure wave; TTI, tension-time index.

All measurements were performed at the same time of the day on the first day of the 7-day training camp and the day after the camp ended. Each subject fasted overnight prior to all measurements. Aortic and brachial hemodynamic parameters were recorded after at least 15 min supine-position rest in a quiet air-conditioned room (24–25°C). Subjects abstained from alcohol for 24 h and caffeine for 12 h prior to the experiment. Electrocardiogram (ECG), left carotid arterial pressure waveforms (via applanation tonometry sensor), and brachial BP (via oscillometric sensors) were simultaneously measured using a vascular function-screening device (form PWV/ABI, Omron-Colin, Kyoto, Japan). Carotid arterial pressure waveforms were transferred into aortic pressure waveforms by pulse wave analysis software involving a validated generalized transfer function (SphygmoCor software, AtCor Medical, Sydney, Australia) (Figure 1). The aortic hemodynamic parameters including aortic systolic BP, pulse pressure, augmentation pressure (AP), augmentation index (Alx), Alx corrected for heart rate at 75 beats per minutes (AIx_75_), time to wave reflection (T_R_), tension time index (TTI), diastolic time index (DTI), and sub-endocardial viability ratio (SEVR) were computed as previously reported (Vlachopoulos et al., 2010).

The 7-day camp was conducted in August (summer season). Although all subject engaged in regular long distance running, the target training distance during the 7-day camp was longer (26 km/day) than the weekly mandatory training distance. The target running distance the week before the camp was 13 km/day. To accomplish such a long distance, subjects participated in three practice sessions per day; 1st session (5:30–7:30), athletes mainly performed long distance running (group, individual, tempo running); 2nd session (8:30–12:30) consisted of jogging, basic core, and lower body strength training; 3rd session (14:00–18:30) consisted mainly of speed development training such as repetition of 400- and 1,000-m sprints. During the camp, all subjects were required to participate all practice sessions, and their schedules were strictly controlled, especially practice duration, meal times, and sleep.

To determine the effect of the intervention on the hemodynamic parameters, repeated measures analysis of variance was performed. In the case of a significant *F*-value, a post hoc test (the Bonferroni method) was performed to identify significant differences in the mean values of interest. For simple correlation analysis as well as to identify the effect of training distance on aortic and brachial BP, three subjects who did not engage any running practice during the camp were excluded due to lower leg injuries. These three subjects trained on the same schedule as others and performed stationary bike training instead of running. Data were reported as the mean ± SD. All comparisons were based on a 95% confidence limit with *P* < 0.05 considered statistically significant.

## Results

**Figure 2:**
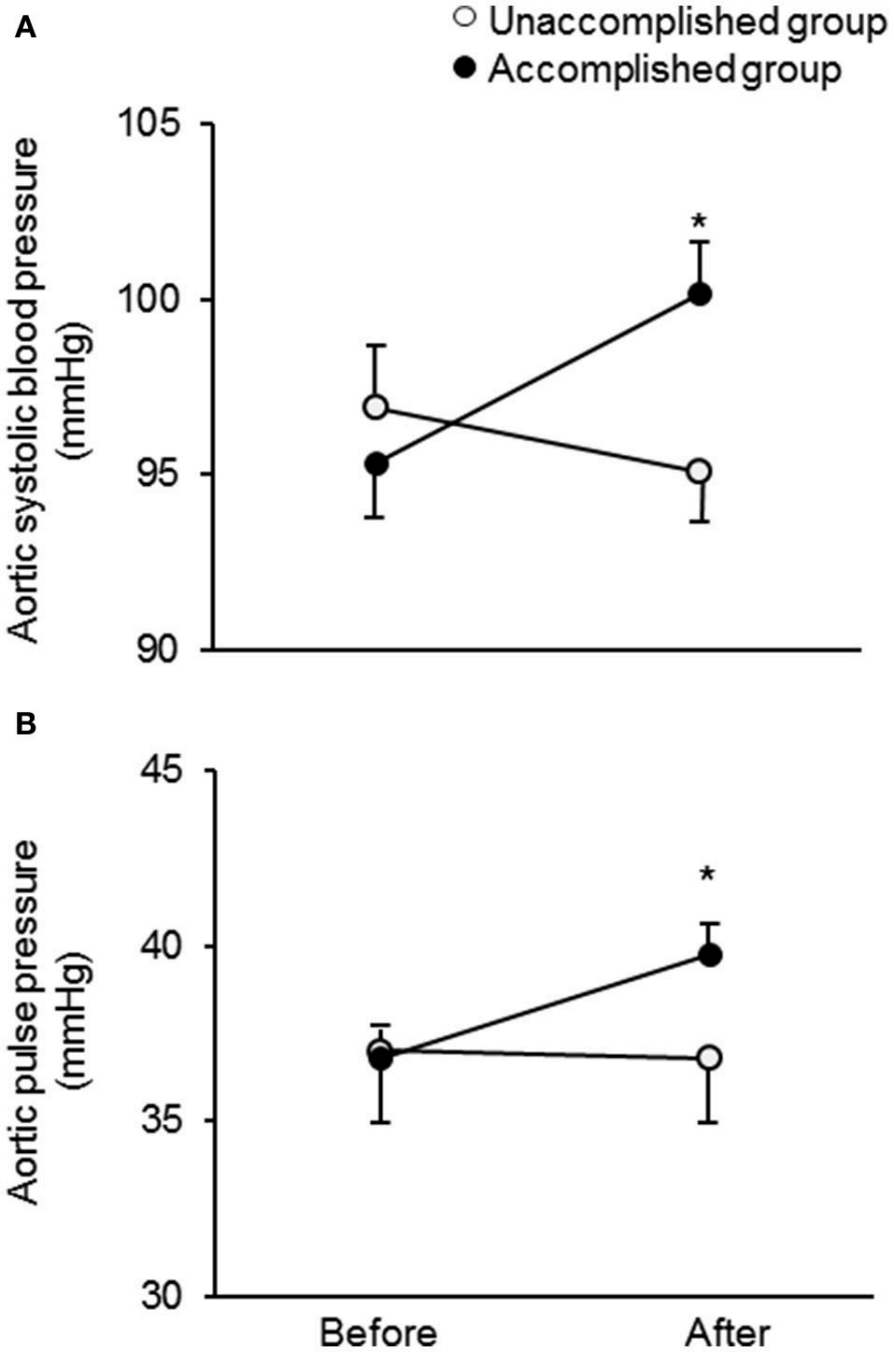
Change in aortic systolic blood pressure **(A)** and pulse pressure **(B)** in the unaccomplished and accomplished groups before and after the camp. ^*^P < 0.05 vs. before the camp.

**Figure 3:**
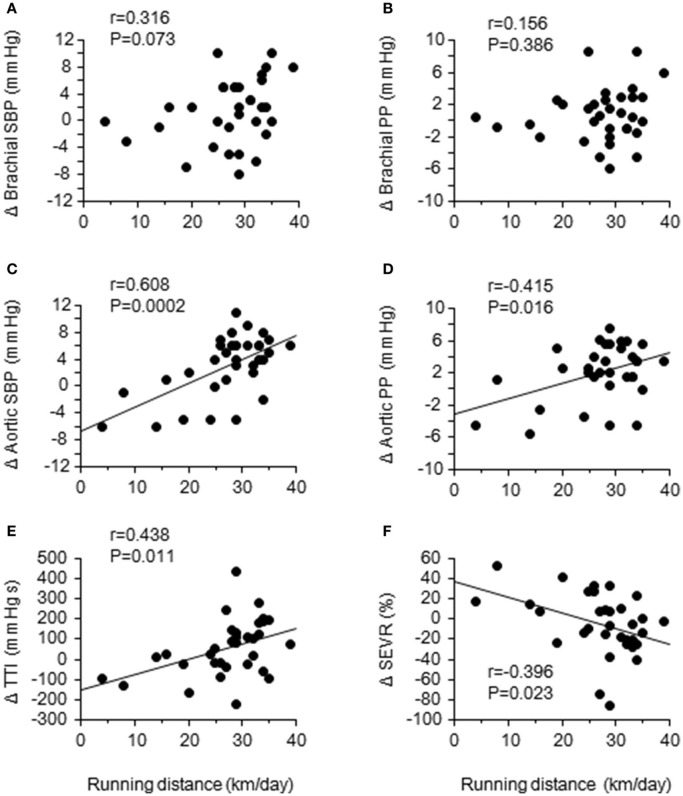
Relationships between the daily training distance and changes in brachial and aortic hemodynamics: brachial and aortic systolic blood pressure (SBP) **(A,C)** and pulse pressure (PP) **(B,D)**; aortic tension-time index (TTI) **(E)**; sub-endocardial viability ratio (SEVR) **(F)**.

After the camp, the athletes were divided into two groups: the accomplished and unaccomplished group. The accomplished group (*n* = 24) achieved the daily and overall target, while the unaccomplished group (*n* = 12) did not complete the training menu due to deconditioning during the camp. The accomplished group constantly completed all training menu, whereas the unaccomplished group mainly engaged to long distance jogging and basic core and lower body strength training without speed development training (e.g. repetition of 400- and 1,000-m sprints). During the camp, the accomplished group completed a longer training distance than the unaccomplished group (31 ± 3 km/day vs. 13 ± 10 km/day, *P* < 0.001). The completed training distance in the accomplished group was approximately 2.5 times longer than 1 week before the camp. Table 1 shows the physical characteristics and hemodynamic parameters before and after the camp. There was no significant group-difference in either physical characteristics or hemodynamic parameters prior to the camp. After the camp, excessive body weight loss, an indicator of dehydration, was not observed. The heart rate, brachial BP, aortic AP, AIx, and AIx_75_ were unchanged in both groups. The aortic systolic BP and pulse pressure significantly increased after the camp in the accomplished group (*P* < 0.001) but not in the unaccomplished group (Figure 2). In the accomplished group, T_R_ shortened and TTI increased significantly (*P* < 0.01), whereas DTI and SEVR did not significantly change after the camp. The average training distance during the camp was not significantly correlated with the changes in brachial systolic BP and pulse pressure, whereas changes in aortic hemodynamic measures were correlated with the average training distance: aortic systolic BP, pulse pressure, TTI, and SEVR (Figure 3). Individual changes in T_R_ did not correlate with corresponding changes in aortic hemodynamic measures: aortic systolic BP (*r* = −0.230, *P* = 0.166); aortic pulse pressure (*r* = −0.055, *P* = 0.750); aortic AP (*r* = 0.012, *P* = 0.945), AIx (*r* = −0.016, *P* = 0.929), and AIx_75_ (*r* = −0.064, *P* = 0.725); TTI (*r* = −0.203, *P* = 0.235); DTI (*r* = 0.056, *P* = 0.746); SEVR (*r* = 0.250, *P* = 0.142).

**Table 1:** Physical characteristics and hemodynamic variables in unaccomplished and accomplished training group before and after the camp.

	**Unaccomplished group (*****n*** = **12)**		**Accomplished group (*****n*** = **24)**	
	**Before**	**After**	**Before**	**After**
Height, cm	171 ± 5	–	171 ± 5	–
Weight, kg	58 ± 5	58 ± 5	58 ± 4	58 ± 4
Heart Rate, bpm	48 ± 5	48 ± 7	47 ± 5	48 ± 5
Brachial systolic BP, mmHg	109 ± 4	109 ± 4	110 ± 8	112 ± 8
Brachial Mean BP, mmHg	76 ± 3	75 ± 3	76 ± 6	78 ± 6
Brachial diastolic BP, mmHg	60 ± 4	58 ± 4	59 ± 6	60 ± 6
Brachial PP, mmHg	49 ± 4	50 ± 5	51 ± 4	52 ± 5
Aortic AP, mmHg	−2 ± 6	0 ± 4	0 ± 5	1 ± 6
AIx, %	−4 ± 12	0 ± 9	−1 ± 11	3 ± 11
AIx_75_, %	−17 ± 13	−13 ± 9	−14 ± 11	−11 ± 11
T_R_, msec	173 ± 10	175 ± 7	172 ± 10	167 ± 11^*^
TTI, mmHg^*^ms	1,399 ± 146	1,366 ± 159	1,342 ± 168	1,434 ± 134^*^
DTI, mmHg^*^ms	3,096 ± 172	3,129 ± 215	3,115 ± 290	3,186 ± 316
SEVR, %	223 ± 27	233 ± 40	235 ± 31	224 ± 27

*Data are mean ± SD. BP, blood pressure; MAP, mean arterial pressure; PP, pulse pressure; AP, augmentation pressure; AIx, augmentation index; AIx_75_, Alx corrected for heart rate at 75 beats per minutes; T_R_, round-trip travel time of the pressure wave from the heart to the peripheral reflected sites; TTI, time tension index; DTI, diastolic tension time integral; SEVR, subendocarial viability ratio*.

* *P < 0.05 vs. before the camp*.

## Discussion

The primary findings from the present study are as flows. First, aortic systolic BP, pulse pressure, and tension-time index (TTI, a surrogate index of the myocardial oxygen demand) were significantly elevated after the camp in the accomplished group but not in the unaccomplished group, whereas the brachial BP remained unchanged in both groups. Secondly, the average daily training distance was significantly correlated with the changes in aortic systolic BP, pulse pressure, and TTI. These results suggest that aortic BP is affected by a short-term vigorous training camp even in highly trained elite endurance athletes partly due to a greater training volume compared to usual.

To the best of our knowledge, this is the first study demonstrating the acute elevation of aortic systolic BP and pulse pressure in elite endurance athletes after short-term endurance training camp characterized by a greater-than-normal training volume. Such changes were associated with a proximally greater than twice longer running distance compared with 1 week before the camp. In addition, individual changes in TTI, a surrogate index of oxygen demand, was positively correlated and SEVR, surrogate indices of myocardial oxygen consumption (Sarnoff et al., 1958) and sub-endocardial perfusion (Buckberg et al., 1972), was correlated negatively with the training distance during the camp. These results suggest that increased training volume with short-term may lead imbalance of myocardial oxygen demand and supply and cause of myocardial fatigue. Our findings could expand the evidence from the cross-sectional investigation that highly trained marathon runners have a higher aortic BP compared with age-matched recreationally active control subjects (Vlachopoulos et al., 2010). More importantly, the training camp did not significantly alter the peripheral BP, and individual changes in peripheral BP were not associated with the training distance during the camp. These results suggest that aortic hemodynamic measures are more sensitive than peripheral BP to acutely increased training volume. To confirm these findings, prospective data linking aortic BP response to chronic overtraining are needed.

The aortic pressure wave may be augmented by overlapping the early return and high amplitude of the reflected wave from the periphery (mainly from lower body) to the proximal aorta on the forward traveling wave generated by the LV ejection in-phase (Nichols and McDonald, 2011). In the present study, T_R_ (a surrogate index of aortic pulse wave velocity) significantly correlated with other aortic hemodynamic measures such as aortic systolic BP and PP. Therefore, increases in aortic systolic BP and PP could not be explained by the early return of the reflected wave from peripheral to the heart. In this context, contrary to the traditional wave theory (Nichols and McDonald, 2011), recent studies suggest that the reflected wave component of arterial hemodynamics does not contribute to augmented central pressure (Schultz et al., 2015; van Mil et al., 2016). Furthermore, it has been also reported that the reflected wave might become diminished until in the proximal aorta, and it no longer possible to identify contributions to central pressure augmentation (Davies et al., 2012). Growing evidence suggests that major determinants of central BP waveform may be the incident waves arising from left ventricular ejection and proximal aortic compliance rather than the wave reflection (Sharman et al., 2009; Davies et al., 2010; Schultz et al., 2014).

The changes in central hemodynamic parameters in this study were potentially impacted by repeated mechanical stress from the heart, inflammatory state from muscle damage, and heart accumulation from prolonged exercise training during summer. First, repeated and particularly excessive stress imposed on the elastic elements of the aortic wall may be a cause of their mechanical fatigue which leads to elevating aortic BP (Nichols and McDonald, 2011). Secondary, aortic BP is affected by inflammatory state common observed in marathon runner after the race (Knez et al., 2006, 2007). It is plausible that the vigorous training-induced muscle damage and systemic inflammation may contribute to elevating aortic BP (Jee et al., 2013). In the present study, the participants of the camp practiced not only on flat track conditions but also on up-and downhill courses; thus, a large amount of exercise stress—especially eccentric contractions during downhill running—was given to the muscle. Furthermore, the 7-day training camp was conducted on summer, thus, the prolonged exercise in the heat causes greater hyperthermia that may yield heat acclimation. Further investigation to clarify the underlying mechanisms for sustained aortic arterial pressure elevation following vigorous intensity exercise training, especially during heat stress, is needed.

Several experimental considerations should be noted. The primary issue is the lack of recorded training intensity, such as heart rate during exercise. For example, by multiplying the duration of a training session by the average heart rate achieved during a session (i.e., training impulse; Macdougall et al., 1982), the training stimulus could be quantified in detail. In addition, we did not evaluate the concomitant changes in biochemical parameters (i.e., inflammation biomarker) and autonomic nervous activity. Measuring these parameters may yield insight on the mechanism of the increased aortic systolic BP after the training camp.

Training programs for highly trained athletes are planned with the repetition in the training cycle composed of intense training periods followed by shorter recovery periods, such as the repetition of over-reaching and super-compensation. The imbalance between training volume/intensity and recovery can lead to an advanced fatigue state (i.e., overtraining syndrome). Therefore, useful (e.g., sensitive) markers to detect fatigue are needed to prevent and manage overtraining for not only athletes but also coaches. Since the strength of this study was a field study with measured hemodynamics parameters in controlled condition among well-trained endurance athletes before and after the camp, the results of this study may provide the consideration of planning short-term summer training camps.

In conclusion, in highly-trained elite endurance athletes, the aortic systolic BP and pulse pressure increases acutely without concomitant elevations in brachial BP after a 7-day training camp characterized by a greater training volume compared with regular training. These alterations might be associated with a greater training volume.

## Author contributions

TT, JS, AH, TI, and SO: Decided conception and design of research; performed experiments; analyzed data; TT, JS, SM, and SO: Interpreted results of experiments; TT and JS: Prepared figures; TT, JS, and SO: Drafted manuscripts; TT, JS, AH, TI, SM, and SO: Approved final version of manuscripts.

### Conflict of interest statement

The authors declare that the research was conducted in the absence of any commercial or financial relationships that could be construed as a potential conflict of interest. The reviewer NF declared a shared affiliation, with no collaboration, with one of the authors, SM, to the handling Editor.

## References

[B1] Buckberg G. D. Towers B. Paglia D. E. Mulder D. G. Maloney J. V. (1972). Subendocardial ischemia after cardiopulmonary bypass. J. Thorac. Cardiovasc. Surg. 64, 669–684. 5083573

[B2] Burr J. F. Drury C. T. Phillips A. A. Ivey A. Ku J. Warburton D. E. (2014). Long-term ultra-marathon running and arterial compliance. J. Sci. Med. Sport 17, 322–325. 10.1016/j.jsams.2013.04.018 23707138

[B3] Davies J. E. Alastruey J. Francis D. P. Hadjiloizou N. Whinnett Z. I. Manisty C. H.. (2012). Attenuation of wave reflection by wave entrapment creates a “horizon effect” in the human aorta. Hypertension 60, 778–785. 10.1161/HYPERTENSIONAHA.111.180604 22802223

[B4] Davies J. E. Baksi J. Francis D. P. Hadjiloizou N. Whinnett Z. I. Manisty C. H.. (2010). The arterial reservoir pressure increases with aging and is the major determinant of the aortic augmentation index. Am. J. Physiol. Heart Circ. Physiol. 298, H580–H586. 10.1152/ajpheart.00875.2009 20008272 PMC2822572

[B5] Halliwill J. R. (2001). Mechanisms and clinical implications of post-exercise hypotension in humans. Exerc. Sport Sci. Rev. 29, 65–70. 10.1097/00003677-200104000-00005 11337825

[B6] Jee H. Park J. Oh J. G. Lee Y. H. Shin K. A. Kim Y. J. (2013). Effect of a prolonged endurance marathon on vascular endothelial and inflammation markers in runners with exercise-induced hypertension. Am. J. Phys. Med. Rehabil. 92, 513–522. 10.1097/PHM.0b013e31829232db 23685440

[B7] Knez W. L. Coombes J. S. Jenkins D. G. (2006). Ultra-endurance exercise and oxidative damage: implications for cardiovascular health. Sports Med. 36, 429–441. 10.2165/00007256-200636050-00005 16646630

[B8] Knez W. L. Jenkins D. G. Coombes J. S. (2007). Oxidative stress in half and full Ironman triathletes. Med. Sci. Sports Exerc. 39, 283–288. 10.1249/01.mss.0000246999.09718.0c 17277592

[B9] Macdougall J. D. Wenger H. A. Green H. J. Canadian Association of Sports, Sciences, Sport Medicine Council of Canada (1982). Physiological Testing of the Elite Athlete. Hamilton, ON: Published by the Canadian Association of Sport Sciences, in collaboration with the Sport Medicine Council of Canada.

[B10] Nichols W. W. McDonald D. A. (2011). Mcdonald's Blood Flow in Arteries Theoretical, Experimental and Clinical Principles, 6th Edn. London: Hodder Arnold.

[B11] Roman M. J. Devereux R. B. Kizer J. R. Lee E. T. Galloway J. M. Ali T.. (2007). Central pressure more strongly relates to vascular disease and outcome than does brachial pressure: the Strong Heart Study. Hypertension 50, 197–203. 10.1161/HYPERTENSIONAHA.107.089078 17485598

[B12] Roman M. J. Okin P. M. Kizer J. R. Lee E. T. Howard B. V. Devereux R. B. (2010). Relations of central and brachial blood pressure to left ventricular hypertrophy and geometry: the Strong Heart Study. J. Hypertens. 28, 384–388. 10.1097/HJH.0b013e328333d228 20051906

[B13] Rowe G. C. Safdar A. Arany Z. (2014). Running forward: new frontiers in endurance exercise biology. Circulation 129, 798–810. 10.1161/CIRCULATIONAHA.113.001590 24550551 PMC3981549

[B14] Sarnoff S. J. Braunwald E. Welch G. H. Jr Case R. B. Stainsby W. N. Macruz R. (1958). Hemodynamic determinants of oxygen consumption of the heart with special reference to the tension-time index. Am. J. Physiol. 192, 148–156. 10.1152/ajplegacy.1957.192.1.148 13498167

[B15] Scharhag J. Herrmann M. Urhausen A. Haschke M. Herrmann W. Kindermann W. (2005). Independent elevations of N-terminal pro-brain natriuretic peptide and cardiac troponins in endurance athletes after prolonged strenuous exercise. Am. Heart J. 150, 1128–1134. 10.1016/j.ahj.2005.01.051 16338248

[B16] Schultz M. G. Davies J. E. Hardikar A. Pitt S. Moraldo M. Dhutia N.. (2014). Aortic reservoir pressure corresponds to cyclic changes in aortic volume: physiological validation in humans. Arterioscler. Thromb. Vasc. Biol. 34, 1597–1603. 10.1161/ATVBAHA.114.303573 24812322

[B17] Schultz M. G. Hughes A. D. Davies J. E. Sharman J. E. (2015). Associations and clinical relevance of aortic-brachial artery stiffness mismatch, aortic reservoir function, and central pressure augmentation. Am. J. Physiol. Heart Circ. Physiol. 309, H1225–H1233. 10.1152/ajpheart.00317.2015 26276816 PMC4632184

[B18] Seals D. R. Desouza C. A. Donato A. J. Tanaka H. (2008). Habitual exercise and arterial aging. J. Appl. Physiol. 105, 1323–1332. 10.1152/japplphysiol.90553.2008 18583377 PMC2576026

[B19] Sharman J. E. Davies J. E. Jenkins C. Marwick T. H. (2009). Augmentation index, left ventricular contractility, and wave reflection. Hypertension 54, 1099–1105. 10.1161/HYPERTENSIONAHA.109.133066 19720955

[B20] Thompson P. D. Buchner D. Pina I. L. Balady G. J. Williams M. A. Marcus B. H.. (2003). Exercise and physical activity in the prevention and treatment of atherosclerotic cardiovascular disease: a statement from the Council on Clinical Cardiology (Subcommittee on Exercise, Rehabilitation, and Prevention) and the Council on Nutrition, physical activity, and metabolism (Subcommittee on Physical Activity). Circulation 107, 3109–3116. 10.1161/01.CIR.0000075572.40158.77 12821592

[B21] Tomoto T. Sugawara J. Hirasawa A. Imai T. Maeda S. Ogoh S. (2015). Impact of short-term training camp on arterial stiffness in endurance runners. J. Physiol. Sci. 65, 445–449. 10.1007/s12576-015-0383-6 26037815 PMC10717420

[B22] van Mil A. C. Pearson J. Drane A. L. Cockcroft J. R. Mcdonnell B. J. Stöhr E. J. (2016). Interaction between left ventricular twist mechanics and arterial haemodynamics during localised, non-metabolic hyperaemia with and without blood flow restriction. Exp. Physiol. 101, 509–520. 10.1113/EP085623 26800643

[B23] Vlachopoulos C. Kardara D. Anastasakis A. Baou K. Terentes-Printzios D. Tousoulis D.. (2010). Arterial stiffness and wave reflections in marathon runners. Am. J. Hypertens. 23, 974–979. 10.1038/ajh.2010.99 20489686

